# Toxicological and histopathological alterations in the heart of young and adult albino rats exposed to mosquito coil smoke

**DOI:** 10.1007/s11356-023-28812-2

**Published:** 2023-07-27

**Authors:** Abeer El-Said Abdrabouh

**Affiliations:** Zoology Department, Faculty of Science, Mansoura University, Mansoura, Egypt

**Keywords:** Mosquito coil, Heart, Enzymes, Inflammatory markers, Hematological parameters, Histopathology, Apoptosis

## Abstract

Mosquito coil repellents are well-known indoor air pollutant with significant health concerns. The present study investigated the toxic effects of mosquito coil smoke on the heart of young and adult male rats. The animals were subjected to the smoke for 6 h/day, 6 days/week, for 4 weeks. Within the first hour after lighting the coil, significant amounts of formaldehyde, total volatile organic compounds, and particulate matter (PM2.5 and PM10) were detected. Both exposed ages, particularly the young group, showed a significant increase in the activities of serum aspartate aminotransferase, lactate dehydrogenase, creatine kinase-MB, and the levels of troponin I, myoglobin, Na^+^ levels, lipid profile, and inflammatory markers (interleukin-6 and C-reactive protein) as well as a significant decrease in K^+^ levels and cardiac Na–K ATPase activity, indicating development of cardiac inflammation and dysfunction. Furthermore, the toxic stress response was validated by significant downregulation at expression of the detoxifying enzyme cytochrome p450. Histopathological studies in both age groups, especially the young group, revealed cardiomyocyte degeneration and necrotic areas. Moreover, upregulation at the pro-apoptotic markers, caspase3, P53, and cytochrome C expressions, was detected by immunohistochemical approach in heart sections of the exposed groups. Finally, the myocardial dysfunctional effects of the coil active ingredient, meperfluthrin, were confirmed by the docking results which indicated a high binding affinity of meperfluthrin, with Na–K ATPase and caspase 3. In conclusion, both the young and adult exposed groups experienced significant cardiac toxicity changes evidenced by cell apoptosis and histopathological alterations as well as disruption of biochemical indicators.

**Graphical Abstract:**

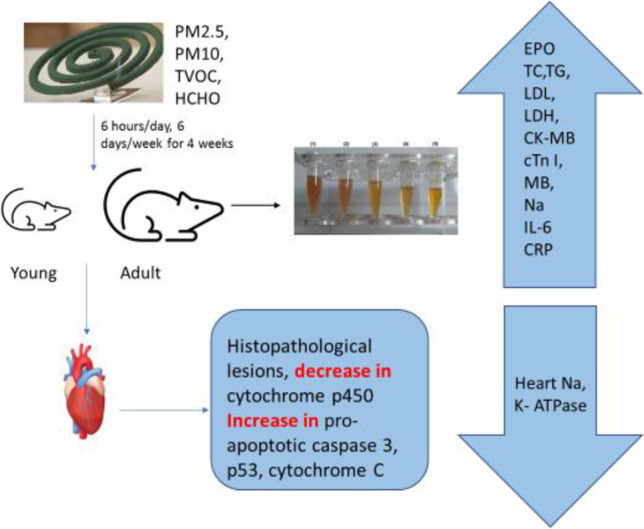

## Introduction

Indoor air pollution is considered among the most prominent driver of environment-induced health risks. The main types of residential insecticide products include aerosols, liquid vaporizers, and mosquito coils. Pyrethrin/pyrethroids are the most widely used active ingredients of mosquito coils that were believed to have low toxicity in humans and untargeted animals (Li et al. 2016). However, recent studies showed adverse effects of these components on human and animal wellness (Hassan et al. 2019; Liu et al. 2022; Badr and Vornanen 2023). Mosquito coils are generally manufactured from materials such as sawdust, coconut shell powder, wrappings, dyes, oxidants such as nitrate, and other additives that control smoldering (Pierre et al. 2017). Combustion of these materials releases insecticide ingredients as well as large amounts of submicrometer particles and gaseous pollutants into the environment (Singh et al. 2020; Elehinafe et al. 2022). Naz et al. (2019) indicated that the gas phase of mosquito coil smoke contains carbonyl compounds including formaldehyde and acetaldehyde. Burning a single mosquito coil has the same bad effects as burning 75–137 cigarettes on health, in addition to the emission of formaldehyde that is equivalent to 51 cigarettes (Uthman et al. 2016; Andini et al. 2022).

A large population is routinely using mosquito coils on daily bases in closed environment to get rid of mosquito biting in complete unawareness of the potential hazard impact, especially during summer (Karim et al. 2020; Anyabolu et al. 2021). Due to the economic affordability and easy market accessibility to this type of mosquito repellent, it is widely used in Asia, South America, and Africa, including Egypt, especially in rural areas (Abdrabouh 2021). Approximately 2 billion people are exposed to elevated smoke resulting from incomplete biomass combustion of 50 billion mosquito coils burned, resulting in indoor air pollution (Syed et al. 2019). Epidemiological studies showed that children and their parents exposed to mosquito coil smoke are more susceptible to the released chemicals overnight during sleeping quarters (Hassan et al. 2019). This may cause respiratory problems (Hogarh et al. 2016) and mutagenic effects (Chen et al. 2008).

The World Health Organization suggested that early exposure to pollutants during childhood and adolescent years can alter the trajectory of the child’s health and increase the incidence of cardiac diseases (World Health Organization 2018). Moreover, Schraufnagel (2020) and Yang et al. (2020) reported that air pollutants could virtually contribute to the onset of cardiovascular diseases even at low levels of exposure. Several biomarkers are effectively used for the diagnosis of cardiac diseases. For instance, liver transaminases, especially aspartate transaminase (AST), was first described in myocardial injury and necrosis in 1954 and remained among the most important biomarkers for several years (Alvarez and Mukherjee 2011; Abdulla Al-Mamun et al. 2017). Creatine kinase-myoglobin binding (CK-MB), which is mostly found in the heart and lactate dehydrogenase (LDH) which plays an important role in the cellular production of energy, are also classical cytosolic enzymes, serving as biomarkers for the diagnosis of myocardial damage. In normal physiological conditions, they are constitutively retained within the endochylema of cardiomyocytes. When the cell membrane becomes permeable or destroyed during myocardial injury, CK-MB and LDH can easily transit through the cytoplasmic membrane into the circulation. Thus, their activity indirectly indicates the extent of myocardial damage (Xu et al. 2020).

Cardiac troponins (cTn) are specific proteins constitute of thin filaments that aggregate to form thick filaments responsible for cardiomyocytes’ contractility (Hammarstena et al. 2018). Due to its cardiac specificity, the cTn is a preferred biomarker for laboratory diagnosis of myocardial infarction (Aydin et al. 2019; Parsanathan and Jain 2020). Moreover, Na–k ATPase is related to the cardiac contractile function either in humans or animals (Yan et al. 2016). Furthermore, exposure to air pollutants may lead to immune suppression, producing an accumulation of immune complexes through different mechanisms, including the uncontrolled release of inflammatory markers (Hamanaka and Mutlu 2018; Kunovac et al. 2020; Hung et al. 2021).

On the other hand, although programmed cell death (apoptosis) is an essential process for cellular and tissue homeostasis, maintaining cell growth and differentiation, and controlling tissue repair, however, excessive heart apoptosis induces cardiac dysfunction (Del Re et al. 2019). Apoptosis and inflammation were assigned as the two major pathological mechanisms of cardiac function deterioration (Sun et al. 2017). Cardiomyocyte apoptosis is a low-abundance process, identified in the diseased heart of humans and rodents. The activation of such process is considered sufficient to lead to heart failure (Bogazzi et al. 2011; Feridooni et al. 2011).

Therefore, the present study aims to investigate the possible toxicological changes at the structure and function of the heart of young and adult male rats upon exposure to mosquito coil smoke using biochemical, histopathological, immunohistochemical, and bioinformatics approaches.

## Materials and methods

### Experimental protocol

Forty male Wistar rats of two ages, young rats aged 3 weeks weighing 75 ± 5 g and adults aged 9 weeks weighing 220 ± 5 g, were purchased from the Egyptian Institute for Serological and Vaccine Production, Helwan, Egypt. The animals were maintained in stainless steel cages at room temperature (25 ± 5 ℃) and humidity 50 ± 5% with a natural day and night cycle. Rats were provided food and water ad libitum under the approval of Animal Care Committee of Mansoura University, Egypt (No. Sci-Z-P-2022–82). After an acclimation period of a week, each age group was randomly subdivided into two groups, each of 10 rats: control young (CN-Y) and exposed young (EX-Y) groups, as well as control adult (CN-A) and exposed adult (EX-A) groups. Control groups inhaled ambient air, while exposed groups were allowed to inhale mosquito coil smoke in an exposure chamber of dimensions 1.5 × 0.9 × 2.1 m^3^ previously described in Abdrabouh (2021) for 6 h (8 am–2 pm)/day, 6 days/week for 4 weeks.

### Mosquito coil characters

A commercially available brand of mosquito repellent coil (Jinjiang Laojun Chemical Co., Ltd, China) with productive approved certificate number HNP35042-I2707 was purchased from retrial outlets at Aga villages, Mansoura, Dakahlia, Egypt, was used in this study. The repellent black coils were declared to contain 0.05% meperfluthrin as an active ingredient. Each coil was approximately 15 cm in diameter, weighed 24–32 g, and was alight for 7–10 h. The same brand of repellent coils was used throughout the study period to avoid variations in commercial products.

### Detection of indoor air quality

DI. ZENE portable air quality detector (model: DZ-8600, USA) was used to detect levels of particulate matter, PM2.5 and PM10 (μg/m^3^), total volatile organic compounds (TVOCs), and formaldehyde (HCHO) mg/m^3^, inside the exposure chamber at zero time and after each hour of the exposure period (6 h/day). Also, the quality of the ambient air in the control chamber was detected.

### Blood sampling

After the experimental period, each animal group was sacrificed under anesthesia by intraperitoneal (I.P.) injection by a mixture of ketamine (0.08 mL/g) and xylazine (0.008 mL/g), where each rat received 0.001 mL/g of this mixture.

Two blood samples were collected per rat. The first sample was pooled in ethylenediaminetetraacetic acid (EDTA) tubes for investigating hematological parameters, including red blood cell (RBCs) count, hemoglobin (Hb) content, hematocrit (HCT)%, platelet (PLT) count, and total white blood cell (WBCs) count, using a fully automatic hematological analyzer (Sysmex XE-2100, Corp., Kobe, Japan), as described by Dacie and Lewis (2001). The second blood sample was centrifuged for 15 min at 855 × g, and the separated serum was preserved at – 20 °C until analysis.

### Serum biochemical studies

In serum samples of each group, erythropoietin hormone (EPO), interleukin-6 (IL-6) as an inflammatory marker, and serum levels of cardiac troponin-I (cTn-I) were determined using the enclosed methods of ELISA kit of Cusabio, Houston, USA, with catalog numbers CSB-E07323r, CSB-E04640r, and CSB-E08594r, respectively. However, the Spinreact Kit, Ctra Santa Coloma, Spain, was used to detect serum levels of C-reactive protein (CRP), lipid profile including total cholesterol (TC), total triglycerides (TG), low- and high-density lipoprotein cholesterols (LDL) and (HDL), as well as aspartate aminotransferase (AST), lactate dehydrogenase (LDH), creatine kinase-MB (CK-MB) enzymes, in addition to Na^+^ and K^+^ ions according to the enclosed method of each parameter. Each with catalog number of MDTLIS40-P, TKBSIS48-E, MXBSIS49-I, MXBSIS51-I, MDBSIS37-I, MDBEIS46-I, TKBEIS43-I, MXBEIS31-I, BSIS54-I, and BSIS53-I, respectively. However, serum myoglobin (MB) was determined according to the included method of BioVision (Milpitas, CA, USA), catalog number E4328-100.

### Heart Na–K ATPase

Half of the heart tissue from each rat was detached to estimate heart Na–K ATPase activity. Approximately 0.5 g of heart tissue was homogenized in phosphate buffer (0.1 M, pH 7.4) and centrifuged at 855 × g for 10 min. Then clear supernatants were collected and frozen at – 80 °C until detection using the method enclosed in the ELISA kit (MyBiosource, Inc., San Diego, USA), catalog number MBS7245054.

### Heart histopathological examinations

The other half of the detached heart tissue was washed through a saline solution, fixed in 10% formaldehyde for 72 h, then dehydrated through an alcohol series, cleared in xylene, embedded in paraffin wax, and cut into sections of 5 μm thickness to be used in histological and immunohistochemical assays.

### Histological studies

Hematoxylin and eosin (H&E) staining was used for evaluation of the heart histopathological alterations. Paraffin sections were cleared in xylene for 2 min, three times, then hydrated by three changes of ethanol (100%, 95%, 70%), 2 min for each concentration. Slides were rinsed by running tap water for 2 min. Heart sections were stained with hematoxylin solution for 3 min, then rinsed with tap water for 5 min. After that, a working eosin solution was applied for 2 min. Dehydration of heart sections was achieved by dipping slides in 95% ethanol for 5 min, then transferred to 100% ethanol twice for 2 min per each. Consequently, heart sections were cleared in xylene 3 times, 2 min per each, and a drop of mounting medium was placed over the slide and covered by a coverslip (Cardiff et al. 2014).

### Histopathological evaluation and semiquantitative scoring

For H&E heart sections in both young and adult groups, the extent of cardiac tissue injury was evaluated via a semi-quantitative scoring system; five randomly selected fields were evaluated for each section in each group. The scoring of cardiac lesion severity depended on the percentage of tissue involvement, as described by Khafaga and El-Sayed (2018): none (0), no involvement of evaluated field; mild (1), involvement of 0–25% of evaluated field; moderate (2), involvement of 25–50% of evaluated field; and severe (3), involvement of 50–100% of the evaluated field.

### Immunohistochemical studies

Five-micrometer-thick positively charged slides were first processed with xylene and alcohol as in histopathological procedures. Antigen retrieval was performed by boiling the samples in 9 mmol/L citrate buffer (PH 6) (Invitrogen, CA, USA) for 30 min. Heart sections were examined for expression of caspase 3, P53, cytochrome C (Cytc) (1:100 dilution; Genemed), and anti-SVV polyclonal antibody (1:75 dilution; Santa Cruz, CA) by overnight incubation with different antibodies at 4 °C. Afterward, slides were incubated with HRP conjugated secondary antibody (1:500 dilution, Santa Cruz, CA) for 1 h (Magaki et al. 2019). Staining with DAB chromogen was conducted using the Vector lab detection kit according to the manufacturers’ instructions (Vector Lab, CA, USA).

For antigen retrieval of the detoxifying enzyme, cytochrome P450 (CYP450), heart tissue slides were placed in antigen retrieval solution (citrate buffer solution, pH 6), then slides were microwaved at power 10 for 5 min two times with adding water if necessary to avoid dryness. Slides were allowed to cool for 15 min, then were washed in deionized water 5 times, and incubated with an endogenous peroxidase blocking reagent containing hydrogen peroxide and sodium azide (DAKO peroxidase blocking reagent, Cat. No. S 2001). One to two drops of the supersensitive primary monoclonal antibody against CYP450 were then put on the sections. Slides were incubated horizontally in humid chamber at room temperature for 60 min. Excess reagent was thrown off and slides were rinsed twice for 5 min in phosphate buffer solution. After blotting excess buffer, 1–2 drops of the ready-to-use DAKO EnVision + system were applied for 20 min at room temperature. Chromogen used was DAB (diaminobenzidine), 1–2 drops for 10–20 min until a desirable brown color was obtained; the slides were then washed in buffer. Sections were taken to distilled water, then nuclear counter staining was done using Mayer’s hematoxylin (Hx) solution for 3–5 min according to degree of nuclear staining. Then, sections were washed in tap water, differentiated in acid-alcohol, and washed again in water. Slides were left to dry in air, then mounted in Canada balsam (Abdraboh et al. 2011).

### Quantitative morphometric measurements

Quantitative morphometric measurements by Leica Quin 500^ image analyzer computer system (Leica image system Ltd.; Cambridge, England) were used to detect the percent of area occupied by expressions of caspase 3, P53, Cytc, and CYP450 in heart sections in each group at a magnification of × 400, according to Shi et al. (2007).

### Molecular docking study of meperfluthrin

The crystal structures were obtained from Protein Data Bank (https://www.rcsb.org/) and meperfluthrin structure from PubChem (https://pubchem.ncbi.nlm.nih.gov/). The docking study was performed using MOE software 2022.02^(1)^, the protein structures were prepared, optimized as well as the ligand structure, and the docking was set to perform 100 runs keeping the top 10 poses for visual inspection.

### Statistical analysis

One-way analysis of variance (ANOVA) followed by Tukey’s post hoc test was used with GraphPad Prism software (v 5.04, GraphPad Software Inc., La Jolla, CA, USA) to statistically analyze the obtained data. The results are expressed as the mean ± standard deviation (SD), and significant values were recorded at *p* < 0.05. The semi-quantitative scoring of cardiac injury parameters was analyzed using Kruskal–Wallis test followed by Dunn’s test to assess the significance between mean scores.

## Results

### Detection of air quality

As seen in Table 1, the portable air quality detector recorded low levels of PM2.5 and PM10 and zero values of TVOC and HCHO at zero time before starting the mosquito coil burn. However, upon ignition of the mosquito coil, PM2.5 and PM10 started to increase from the first hour of exposure to severely polluted limits (> 250 µg/m^3^ and 420 μg/m^3^), respectively. Additionally, TVOCs and HCHO exhibited heavily polluted categories (> 5.00 mg/m^3^ and 0.500 mg/m^3^), respectively. However, at the second hour of exposure, emissions increased, where PM2.5, PM10, and TVOCs recorded stable values of maximal readings, while HCHO increased with time, indicating heavily polluted ambient air. It should also be mentioned that the ambient air in the control chamber was of good quality.

**Table 1:** Indoor air quality in the control and exposure chambers during the experimental period

Time	PM2.5 (ug/m^3^)	PM10 (ug/m^3^)	TVOC (mg/m^3^)	HCHO(mg/m^3^)
Zero	30	38	Nil	Nil
1 h	579	637	7.759	1.345
2 h	999	999	9.999	2.087
3 h	999	999	9.999	2.112
4 h	999	999	9.999	2.874
5 h	999	999	9.999	3.563
6 h	999	999	9.999	3.859
Control chamber	30 ± 3	45 ± 3	0.4 ± 0.1	0.05 ± 0.01
Air quality standards	0–35, good 35–75, moderate 75–115, lightly polluted 115–150, moderately polluted 150–250, heavily polluted > 250, severely polluted	0–50, good 50–150, moderate 150–250, lightly polluted 250–350, moderately polluted 350–420, heavily polluted > 420, severely polluted	0–0.6, good 0.6–2.0, lightly polluted 2.0–5.0, moderately polluted > 5.0, heavily polluted	0–0.08, good 0.08–0.30, lightly polluted 0.30–0.50, moderately polluted > 0.50, heavily polluted

### Serum biochemical studies

The obtained data showed significantly increased levels of EPO hormone, TC, TG, LDL, AST, LDH, CK-MB, cTnI, MB, and Na^+^ along with significantly decreased levels of K^+^ in both exposed young and adult groups compared to their respective control groups. However, HDL levels were non-significantly decreased in both exposed ages. The investigated serum biochemical parameters showed no significant difference between young and adult exposed groups, except for LDH and CK-MB levels. However, the latter was also significantly decreased in the control adult group compared to its respective young group. The % change was more remarkable in TC, LDH, cTnI, Na^+^, and K^+^ in the serum of exposed young group (43.74%, 116.04%, 92.61%, 98.52%, and − 33.21%) compared to the exposed adult (37.75%, 91.72%, 74.51%, 71.28%, and − 25.45%), respectively. However, the adult exposed group showed an obvious increase in the % change for EPO, TG, LDL, HDL, CK-MB, and MB (123.53%, 94.65%, 84.49%, − 21.40%, 103.35%, and 106.40%) compared to the young exposed group (85.71%, 83.96%, 63.98%, − 7.33%, 70.03%, and 87.86%), respectively (Table 2).

**Table 2:** Serum biochemical parameters in the studied young and adult groups

Group	CN-Y	EX-Y	CN-A	EX-A
Parameter				
EPO (ng/mL)	0.133 ± 0.03	0.247^a^ ± 0.08 85.71%	0.102 ± 0.03	0.228^b^ ± 0.02 123.53%
T C (mg/dL)	97.4 ± 13.3	140^a^ ± 10.0 43.74%	95.1 ± 11.5	131^b^ ± 8.71 37.75%
T G (mg/dL)	31.8 ± 4.21	58.5^a^ ± 7.28 83.96%	46.0 ± 7.60	86.7^b^ ± 25.5 94.65%
LDL (mg/dL)	60.8 ± 9.82	99.7^a^ ± 11.4 63.98%	45.8 ± 9.02	84.5^b^ ± 12.0 84.49%
HDL (mg/dL)	40.9 ± 4.54	37.9 ± 8.52 − 7.33%	48.6 ± 3.42	38.2 ± 4.97 − 21.40%
AST (U/L)	138 ± 18.2	194^a^ ± 17.2 40.58%	124 ± 15.1	174^b^ ± 12.3 40.32%
LDH (U/L)	1147 ± 315	2478^a^ ± 351 116.04%	833 ± 88.9	1597 ^bd^ ± 260 91.72%
CK-MB (U/L)	951 ± 89.1	1617^a^ ± 252 70.03%	568 ± 126^c^	1155 ^bd^ ± 242 103.35%
cTn I (pg/mL)	0.176 ± 0.021	0.339^a^ ± 0.036 92.61%	0.153 ± 0.020	0.267^b^ ± 0.049 74.51%
MB (ng/mL)	0.140 ± 0.009	0.263^a^ ± 0.061 87.86%	0.125 ± 0.019	0.258^b^ ± 0.051 106.40%
Na^+^ (mmol/L)	67.5 ± 11.2	134^a^ ± 7.31 98.52%	75.9 ± 6.24	130^b^ ± 13.9 71.28%
K^+^ (mmol/L)	5.36 ± 0.256	3.58^a^ ± 0.532 − 33.21%	5.54 ± 0.647	4.13^b^ ± 0.588 − 25.45%

Values represented as mean ± SD, *CN*, control; *EX*, exposed; *Y*, young; *A*, adult. a: significance between EX-Y and CN-Y; b: between EX-A and CN-A; c: between CN-Y and CN-A; d: between EX-Y and EX-A. % change = [(EX − CN)/CN] × 100

**Fig. 1:**
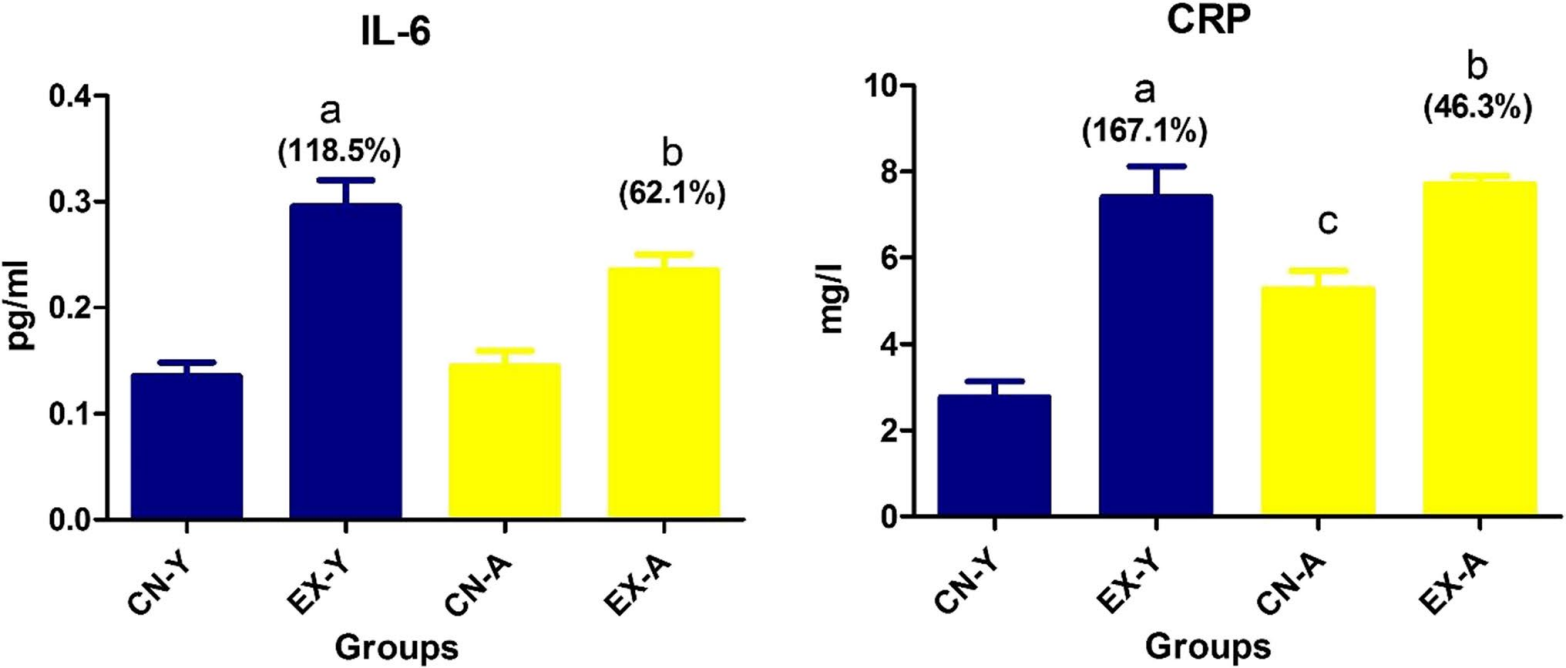
Serum inflammatory markers in the studied young and adult groups. **a**, Significance between EX-Y and CN-Y; **b**, between EX-A and CN-A; **c**, between CN-Y and CN-A; **d**, between EX-Y & EX-A. % change = [(EX − CN)/CN] × 100

The present study also showed a significant increase in serum inflammatory markers (IL-6 and CRP) in both young and adult groups exposed to mosquito coil smoke compared to their controls. This increase was more pronounced in the exposed young group, where the percentages of increases in IL-6 and CRP in the young group were 118.5% and 167.1%, while in the adult exposed group were 62.1% and 46.3%, respectively (Fig. 1). The present data also showed no significant difference in either inflammatory marker between the two exposed ages. Moreover, IL-6 showed a nonsignificant difference between the control group of both studied ages. However, CRP levels in the adult control group increased significantly compared to the control young group.

### Heart Na–K ATPase

**Fig. 2:**
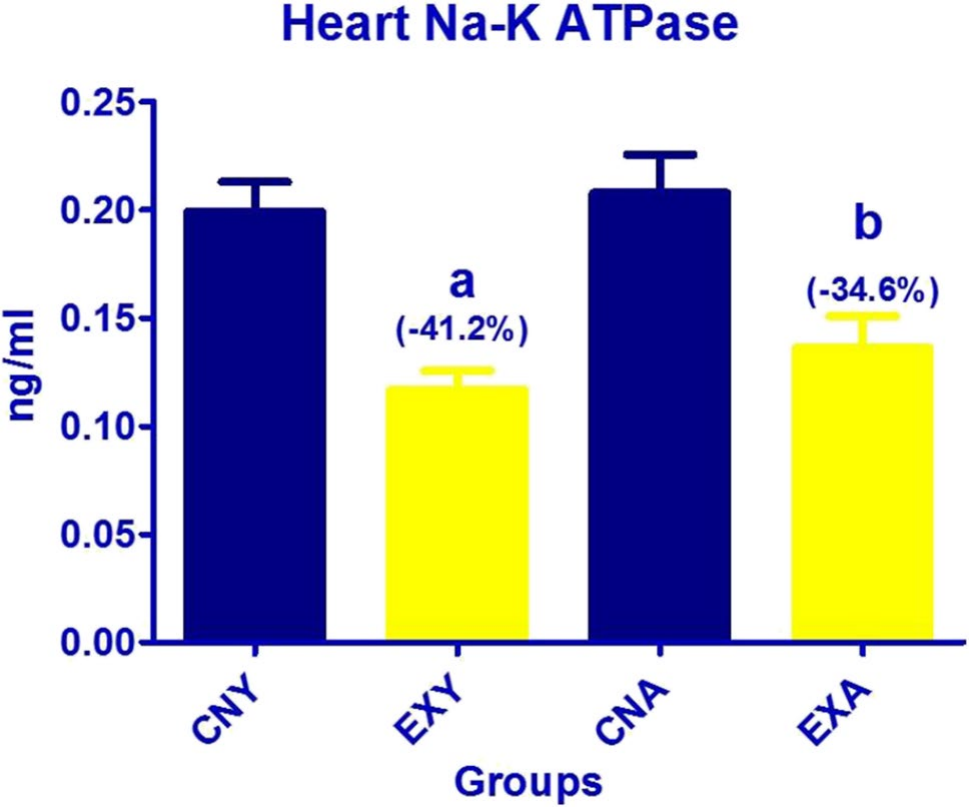
Heart Na–K ATPase in the studied young and adult groups. **a**, Significance between EX-Y and CN-Y; **b**, between EX-A and CN-A; **c**, between CN-Y and CN-A; **d**, between EX-Y and EX-A. % change = [(EX − CN)/CN] × 100

The levels of Na–K ATPase in the heart of the investigated groups exhibited a significant decrease at both exposed ages compared to the control. However, the % change was higher in the young-exposed group than in the adult-exposed group. It should also be mentioned that no significant difference was recorded either between the exposed groups or the control groups of both ages (Fig. 2).

### Hematological parameters

As shown in Table 3, exposure to mosquito coil smoke for 4 weeks exhibited a non-significant increase in hematological parameters, including RBC count, Hb content, and HCT% along with a significant decrease in total WBC count in both the young and adult exposed groups compared to their respective controls. However, the total PLT count was significantly elevated in the young exposed group, but nonsignificant in the adult group. RBC count in the exposed adult group was significantly elevated compared to the exposed young group. The percentage of change (% change) between exposed young and adult groups exhibited some increase in RBC count in the adult exposed group (10.22%) compared to the young exposed group (4.21%). However, the % change in HCT% and total PLT count was increased in the young exposed group (5.68%, 23.06%) compared with the adult exposed group (0.51%, 13.17%), respectively.

**Table 3:** Hematological parameters in the studied young and adult groups

Groups	CN-Y	EX-Y	CN-A	EX-A
Parameters				
RBCs (10^6^/µL)	6.41 ± 0.460	6.68 ± 0.487 4.21%	6.85 ± 0.539	7.55^d^ ± 0.377 10.22%
Hb (g/dL)	12.8 ± 0.731	13.5 ± 0.797 5.47%	13.1 ± 1.36	13.7 ± 0.915 4.58%
HCT (%)	37.0 ± 3.02	39.1 ± 0.877 5.68%	39.3 ± 3.53	39.5 ± 3.66 0.51%
WBCs (10^3^/µL)	10.2 ± 0.907	6.95^a^ ± 1.26 − 31.86%	11.8 ± 2.56	8.29^b^ ± 0.954 − 29.75%
PLTs (10^3^/µL)	503 ± 65.3	619^a^ ± 80.2 23.06%	501 ± 54.8	567 ± 47.6 13.17%

Values represented as mean ± SD. *CN*, control; *EX*, exposed; *Y*, young; *A*, adult. a: significance between EX-Y and CN-Y, b: between EX-A and CN-A, c: between CN-Y and CN-A, d: between EX-Y and EX-A. % change = [(EX − CN)/CN] × 100

### Heart histopathological examination

#### Histological studies

**Fig. 3:**
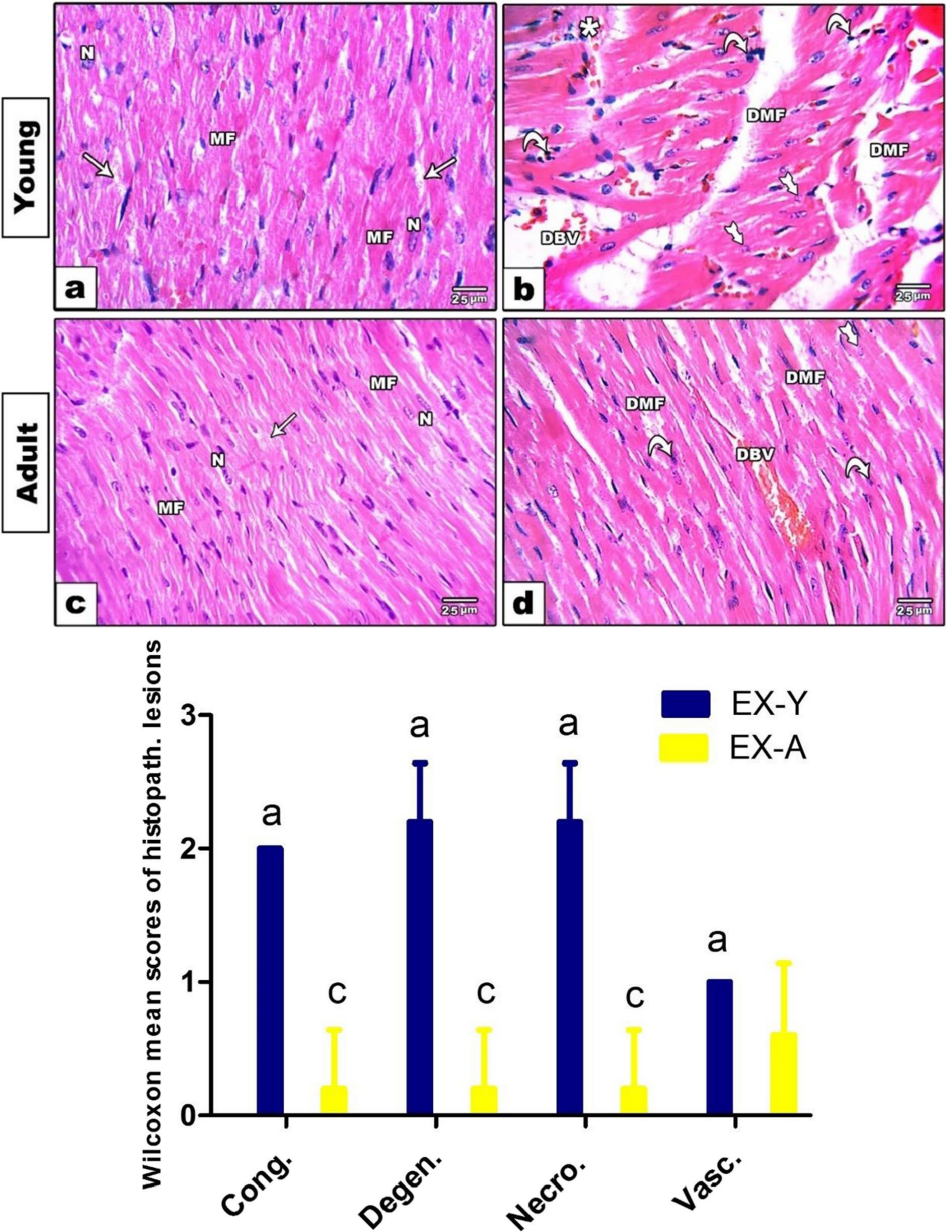
Photomicrographs of longitudinal sections of cardiac muscles stained by H&E, showing young and adult control groups (**a**, **c**) with normal branched striated cardiomyocytes with normal muscle fibers (MF) and intact intercalated discs containing connective tissue and blood vessels (arrow), acidophilic sarcoplasm, and centrally located nuclei (N). Both the exposed young and adult groups (**b**, **d**) showed disarrangement of cardiomyocytes with degenerated muscle fibers (DMF), dilated and congested blood vessels (DBV), pyknotic nuclei (curved arrow), necrotic areas (tailed arrow), and infiltration zones (star), especially in the heart of exposed young group. e Semi-quantitative scoring of heart injury in young and adult rats using Kruskal–Wallis test followed by Dunn’ test to compare all means. Letters a, b, and c means significant difference when *P* < 0.05; **a**, between CN-Y and EX-Y; **b**, between CN-A and EX-A; **c**, between Ex-Y and Ex-A

The heart of the control groups in both young and adult ages stained by H&E showed regular striation and normally branched cardiomyocytes with intact intercalated discs and acidophilic sarcoplasm with centrally located single or binucleus (Fig. 3a, c). In contrast, both exposed ages, especially young group, showed disarrangement of cardiomyocytes characterized by degenerated muscle fibers, dilated, and congested blood vessels. This was accompanied by marked cytoplasmic vacuolation, pyknotic nuclei, and distinguished necrotic spots infiltrated by inflammatory cells (Fig. 3b, d). Heart sections from both young and adult control groups appeared with no lesions (no involvement of evaluated field). However, the heart of young exposed group was involved in mild vasculitis, moderate to severe degeneration, and necrosis, in addition to moderate congestion. The adult exposed group showed less involvement in histopathological lesions, appeared in mild vasculitis, degeneration, necrosis, and congestion in heart tissue (Fig. 3e). Statistically all these changes were significantly increased in young exposed rats compared to adults.

### Immunohistochemical studies

**Fig. 4:**
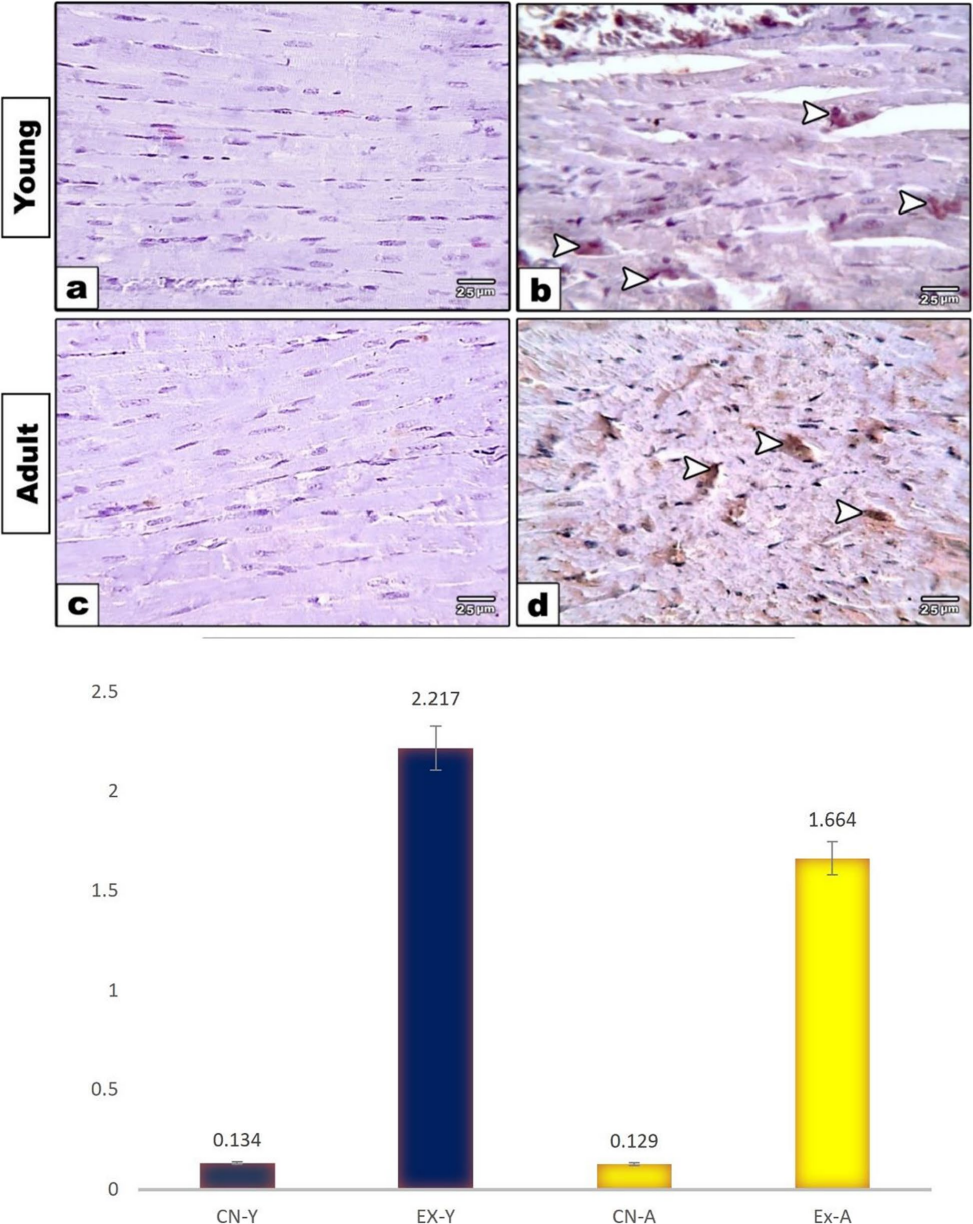
Photomicrographs of heart sections immunostained with caspase 3. Both the young and adult control groups (**a**, **c**) showed negative staining in cardiomyocytes. Exposed young and adult groups (**b**, **d**) showed positive brown expression in cardiomyocytes (arrowheads) that is markedly expressed in young-exposed rats. **e** Histogram showing the percent of caspase 3 in the cardiac muscle fibers of rat groups using image analysis

**Fig. 5:**
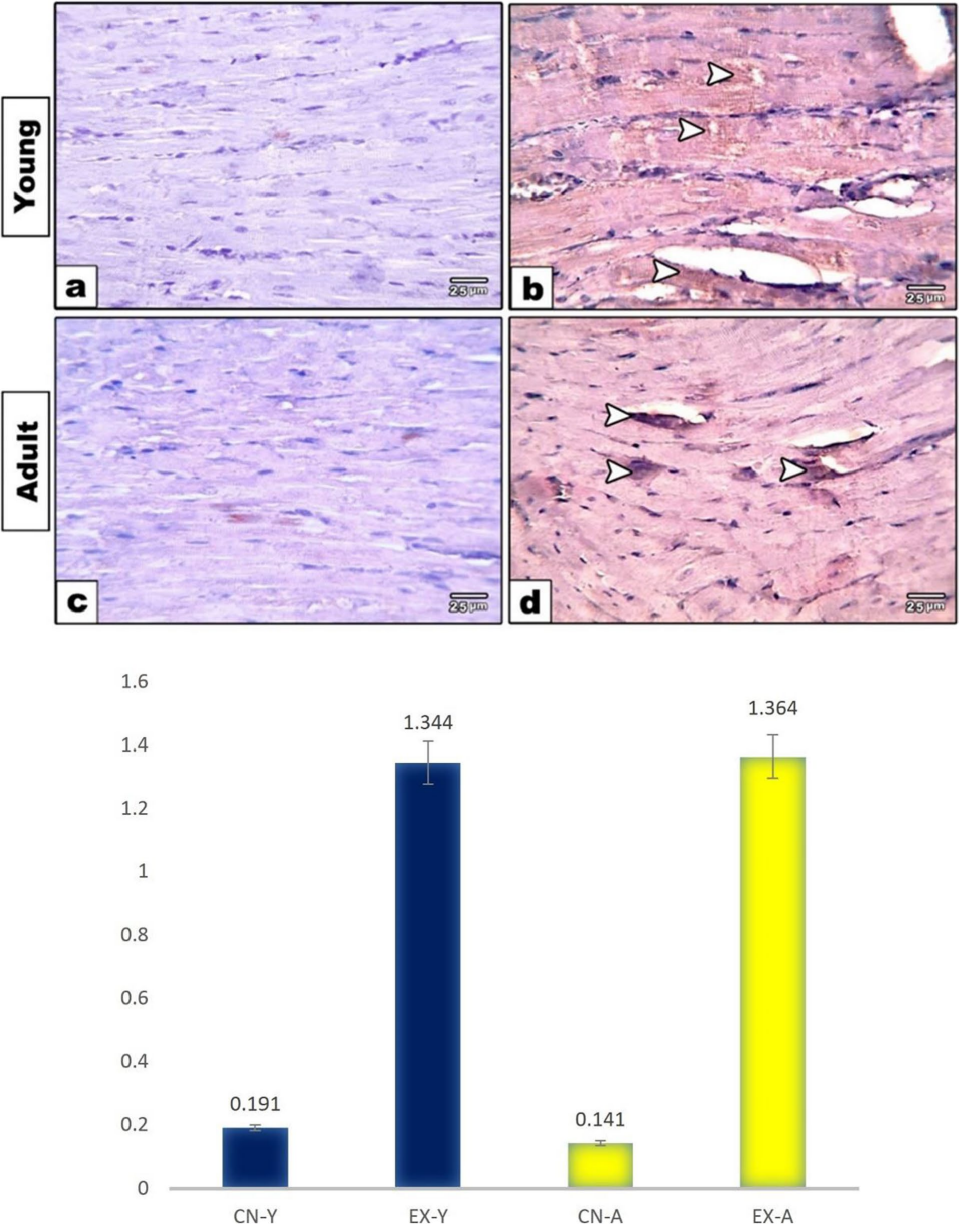
Photomicrographs of heart sections immunostained with P53. Both the young and adult control groups (**a**, **c**) showed mild positive brown staining in cardiomyocytes (arrowheads). Exposed young and adult groups (**b**, **d**) showed increased positive brown expression in cardiomyocytes (arrowheads). **e** Histogram showing the percent of P53 in the cardiac muscle fibers of rat groups using image analysis

**Fig. 6:**
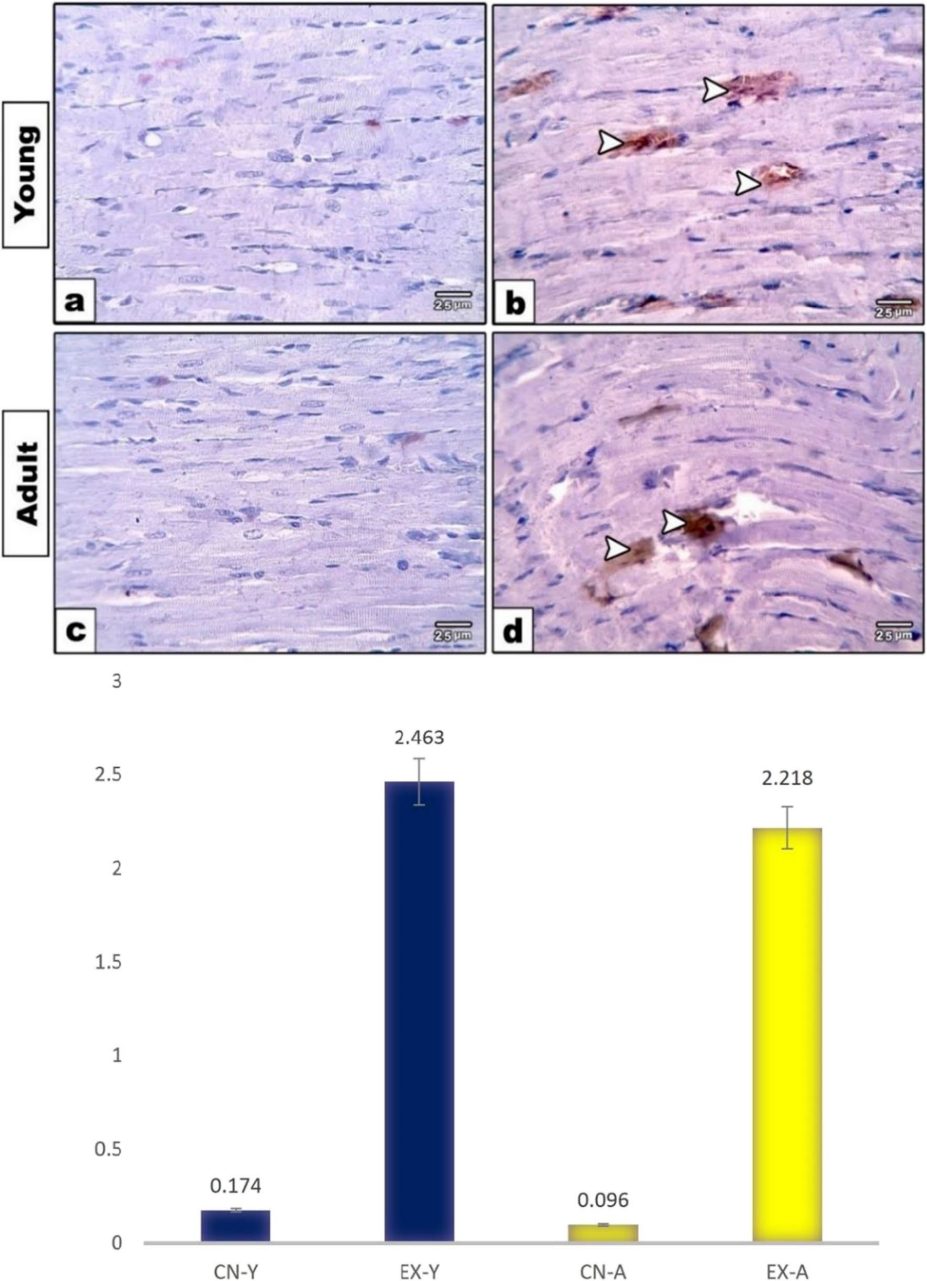
Photomicrographs of heart sections immunostained with Cytc. Both the young and adult control groups (**a**, **c**) showed negative staining in cardiomyocytes. Exposed young and adult groups (**b**, **d**) showed positive brown expression in cardiomyocytes (arrowheads). **e** Histogram showing the percent of cytochrome C in the cardiac muscle fibers of rat groups using image analysis

The expressions of caspase 3, P53, and Cytc in cardiomyocytes of young and adult groups were evaluated. The heart sections immunostained with caspase 3 showed both the young and adult control groups with negatively stained cardiomyocytes (Fig. 4a, c). However, exposed young and adult groups showed positive expression in cardiomyocytes that was more markedly expressed in the young exposed group (Fig. 4b, d). Quantitative morphometric measurements using image analysis supported these observations, where the percent area of caspase 3 expression was increased in the young exposed group (2.217%) compared to the adult (1.664%) (Fig. 4e). However, the respective control groups were 0.134% and 0.129%, respectively. The heart sections immunostained with P53 in both young and adult control groups showed mild reactivity in cardiomyocytes (Fig. 5a, c), while exposed young and adult groups showed increased positive P53 expression (Fig. 5b, d). This was more illustrated through Fig. (5e), where morphometric measurements showed the percent area of P53 expression increased in young and adult exposed groups (1.344% and 1.364%) compared to respective control groups (0.191% and 0.141%). Moreover, a negative immune reaction of Cytc was found in cardiac muscle fibers of the control group of both studied ages (Fig. 6a, c). However, positive expression of Cytc was observed in both the exposed groups (Fig. 6b, d). Image analysis showed the percent area of such positivity as 2.463% and 2.218% in young and adult exposed groups compared to control groups (0.174% and 0.096%), respectively (Fig. 6e).

**Fig. 7:**
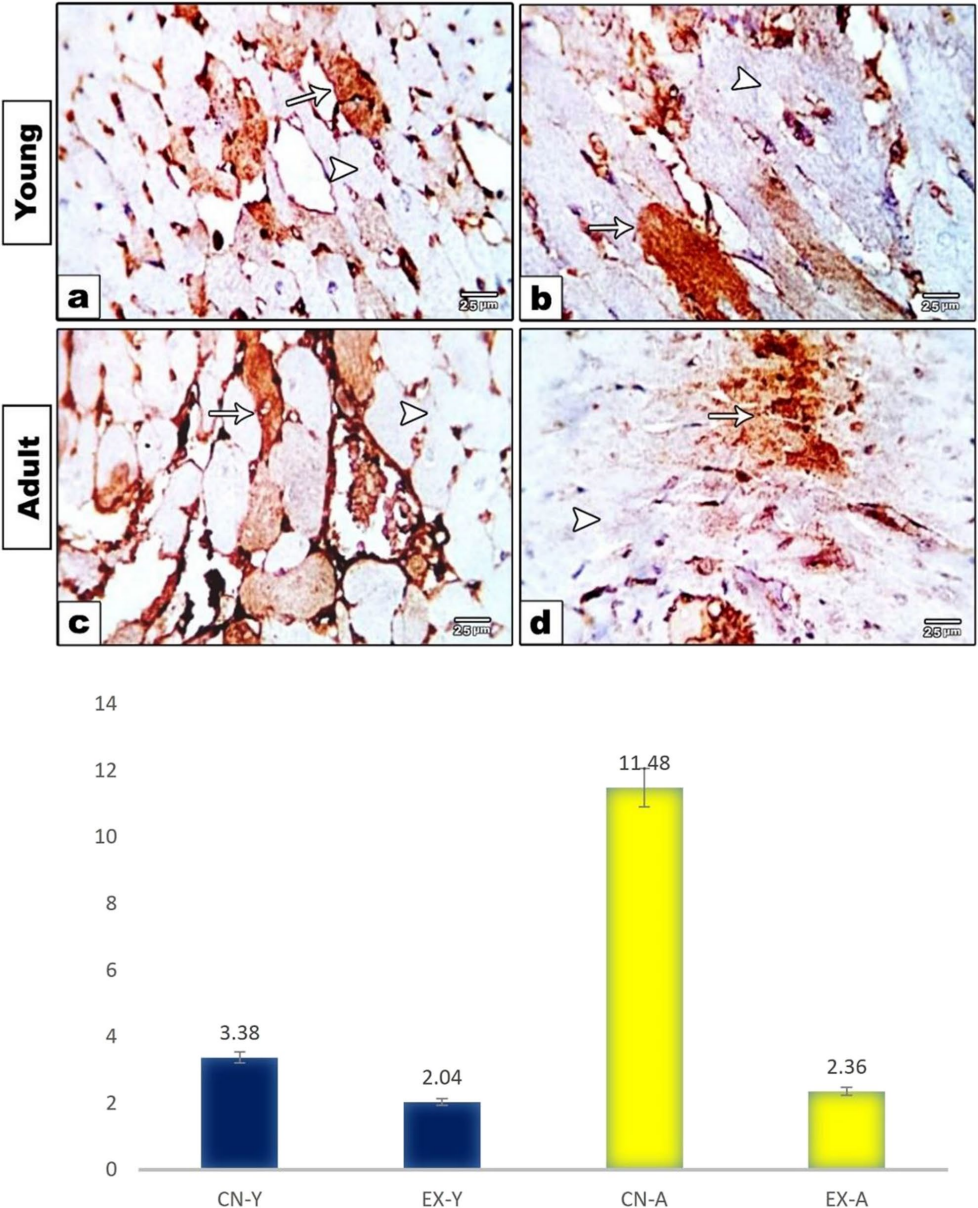
Photomicrographs of heart sections immunostained with CYP450 showing positive brownish cytoplasmic reactivities in different experimental groups. Control young and adult groups showed higher expression (**a**, **c**); meanwhile, exposed young and adult groups showed the least reactivities (**b**, **d**). Positive cardiomyocytes are indicated by arrow and negative cells are indicated by arrowhead. e Histogram showing the percent area of CYP450 in the cardiomyocytes of rat groups using image analysis

Heart tissues in control young group showed few numbers of cardiomyocytes with a positive brownish cytoplasmic reactivity to CYP450 indicated by a moderate to marked positively stained sarcoplasm, sarcolemmal membrane, and occasionally vascular endothelial cells (Fig. 7a). Comparatively, an increased number of cells with positive brownish cytoplasmic staining reaction were recorded in the control adult group (Fig. 7c). However, expressions of positive immuno-stained cardiomyocytes in both young and adult exposed groups were lower as compared with their respective control groups with few moderately reactive cells (Fig. 7b, d). Quantitative morphometric measurements using image analysis supported these observations, where the percent area of CYP450 expression was decreased in the young control group (3.38%) compared to the control adult group (11.48%). However, the expressions of CYP450 in exposed young and adult groups were 2.04% and 2.36%, respectively (Fig. 7e).

### Molecular docking study of meperfluthrin

#### With Na–K pump (Na–K ATPase)

**Fig. 8:**
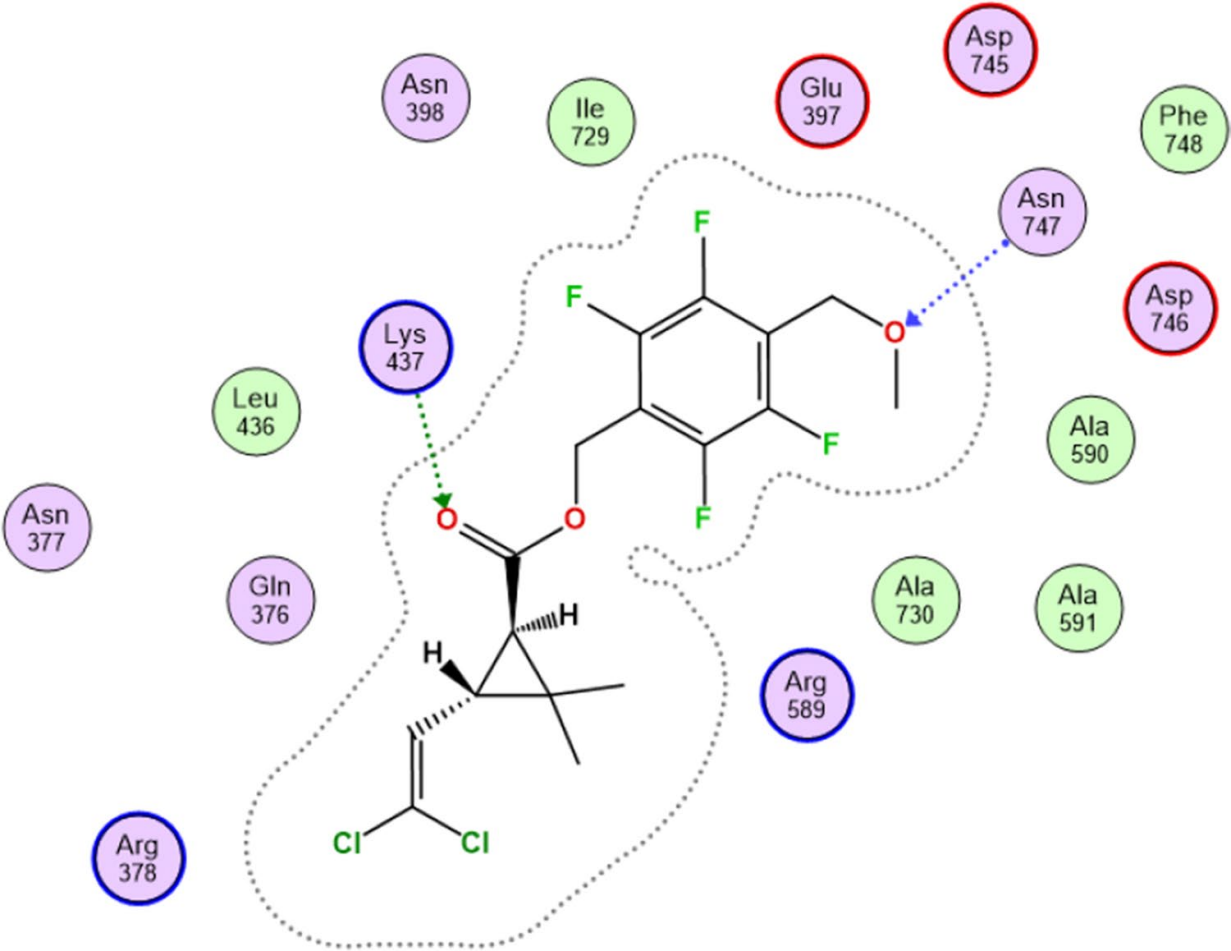
Meperfluthrin showed good binding with sodium–potassium pump (Na + /K + -ATPase, PDB: 3KDP)^(^^4)^

Meperfluthrin showed good binding with sodium–potassium pump (Na/K-ATPase, PDB: 3KDP)^(4)^ where it formed two hydrogen bonds with Lys437, which is involved in substrate recognition and binding, and helps to stabilize the transition state during transport, and Asn747. The binding score was − 6.57 (Fig. 8).

### With Caspase-3

**Fig. 9:**
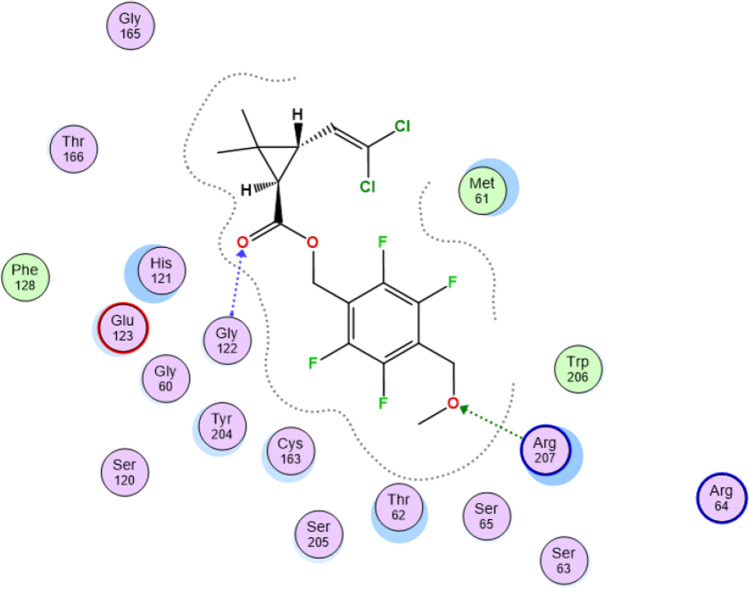
Meperfluthrin showed good binding with the pocket of caspase-3 (PDB: 5I9B)^(^^2)^ with a binding score of (− 6.011) through two hydrogen bonds with Gly122

As shown in Fig. 9, meperfluthrin showed good binding with the pocket of caspase-3 (PDB: 5I9B)^(2)^ with a binding score of − 6.011 through two hydrogen bonds with Gly122, which is important for the flexibility of the active site and Arg207, which is essential in the ligand recognition and binding.

## Discussion

Pyrethroids are extensively used worldwide as commercial and household insecticides (Liu et al. 2022). Continuous inhalation of mosquito coil smoke containing pyrethroid may harm the hearts of both young and adults. The mosquito coils used in the present study were declared to contain meperfluthrin, as one of pyrethroids. However, the air quality assessment recorded significant elevation at the levels of PM2.5, PM10, TVOCs, and HCHO contaminants which were emitted with mosquito coil smoke within the first hour of exposure, indicating rapidly polluted ambient air. This finding supported the study of Kumar et al. (2014), Taylor et al. (2017), and Singh et al. (2020) who attributed this to the aromatization process of organic (base) material involved in the burning process, which included a complex reaction result in different emissions. Other studies attributed the emissions of the mosquito coils to the binding materials which include carbon and other various compounds (Hogarh et al. 2018; Wang et al. 2018; Karim et al. 2020). Furthermore, other findings indicated that burning one mosquito coil might release a comparable amount of PM2.5 resulting from burning 75–137 cigarettes and emitting formaldehyde equivalent to that released from burning 51 cigarettes (Hassan et al. 2019; Andini et al. 2022). On the other hand, Nascimento et al. (2017) revealed that pyrethroids and other hazardous materials can be absorbed on particulate matter and become hazardous through inhalation.

For a future effective therapy, a well-defined understanding of the molecular mechanisms behind the negative effects brought on by exposure to pyrethroids is required. The current study showed sever heart dysfunction manifested by disrupted heart function parameters, altered histological architecture of heart tissue, and decreased CYP 450 in young and adult exposed rats. These effects were coupled with increased inflammatory indicators in the serum of the same animals.

Recent study reported that regular exposure to a common home insecticide may increase the risk of cardiovascular disease and early mortality (Xue et al. 2021). The present study showed extensive cardiac histopathological alterations with increased activities of AST, CK-MB, and LDH as well as troponin I levels in the serum of young and adult animals exposed to mosquito coil smoke for 4 weeks compared to the controls. This suggests that myocardial injury was developed due to a damage of the cardiomyocytes and upregulation of the enzyme level. These results supported the earlier research findings that illustrated the chronic exposure to pyrethroid induced heart remodeling and impairment through oxidative stress, suggesting a possible positive association between pyrethroid exposure and the risk of coronary heart disease (Han et al. 2017; Marques et al. 2022). Additionally, AbouZied et al. (2021) related the elevation in CK-MB to hypoxic stress as an adaptive factor in cases of pathophysiologic myocardial hypoxia. In support, Juhász et al. (2022) elucidated that the increase in myocardial enzymes in serum was linked to a certain extent of myocardial damage after exposure to organic compounds.

Furthermore, MB is one of the cytoplasmic heme proteins, which is used for storing intracellular oxygen and facilitating the distribution of oxygen to the mitochondria in the energy formation process (Herawati et al. 2018). The obtained data revealed a significant elevation in MB levels in both exposed young and adult groups compared to the control groups. Levett et al. (2011) attributed the increase in MB to regulate the blood flow to the heart during hypoxia.

Since the changes in cellular ion fluxes and inhibition of membrane-bound ATPases can alter myocardial contractility, the effect of pyrethroid on cardiac tissue was examined. The current study showed a significant inhibition of Na–K ATPase in the heart and marked disturbance of Na^+^ and K + ions in the pyrethroid-exposed rats. This effect might change the transmembrane ion transport with increased Na^+^ influx, leading to changed membrane potential which in turn causes enhancement of catecholamine release leading to increased myocardium contraction and development of heart dysfunction. Furthermore, the molecular docking data in the present study may explain the significant decrease in Na–K ATPase activity in both young and adult exposed rats through high binding affinity with meperfluthrin.

Moreover, young and adult animals exposed to pyrethroid showed a significant increase in serum lipid fractions (TC,TG, and LDL) compared to the control groups, confirming recent study in fish (Bej et al. 2021) indicating dyslipidemia due to pyrethroid exposure. This might be attributed to the established links between hyperlipidemia and development of atherosclerosis, and the prevalence of cardiovascular diseases (Bhakkiyalakshmi et al. 2016). Taiwo et al. (2008) reported the inability of the mitochondria to utilize cholesterol, phospholipids, and fatty acids for ATP production, causing fatty degeneration of parenchymal cells. This could explain histological alterations observed in the H&E-stained heart sections, especially in young exposed rats compared to the control group. These results were in accordance with other studies that demonstrated histopathological changes in different organs, including heart and lung of rats, exposed to mosquito coil smoke (Taiwo et al. 2008; Andini et al. 2022).

To further disclose the mechanism implicated in disruption of heart structure and function after exposure to pyrethroid, apoptotic regulating proteins were evaluated. The implication of meperfluthrin in cell apoptosis was first validated using molecular docking approach which revealed a high affinity of meperfluthrin for binding with caspas-3. The current study showed induction of myocardial cell apoptosis in exposed groups which pointed out by elevation of pro-apoptotic markers, including caspase 3, P53, and Cytc in both exposed groups compared to control groups. The same observation was obtained by Urich et al. (2009) after exposing mice to PM 2.5. This effect was related to the induction of mitochondrial oxidative stress which activates P53 in the intrinsic apoptotic pathway (Reed et al. 2015; Liu et al. 2019). In this concern, Kelley et al. (2013) suggested that deleterious effects of reactive oxygen species (ROS) may be also induced by inhibiting the expression of CYP450 and compromising the ability of an organism to metabolize and eliminate xenobiotics (Andreau et al. 2012). This response was validated in the present study by significant downregulation at expression of the detoxifying enzyme CYP450 in the heart of exposed groups. This agreed with Jamieson et al. (2017) who suggested that the exposure to chemicals and other environmental agents can independently alter the expression and activity of CYP450 in the detoxification process. On the other hand, the elevated expression of P53 after stress exposure could activate caspase 3 in cardiomyocytes via induction of Cytc release from mitochondria during heart failure (Birks et al. 2008).

Recently, de Oliveira et al. (2020) added that the increase in apoptotic cells may be related to the increase at cell inflammation. The present study showed a marked increase in the serum levels of inflammatory markers, including IL6 and CRP, in young and adult exposed groups after exposure to mosquito coil smoke. These findings agreed with Castrogiovanni et al. (2016), Schraufnagel (2020), and Hung et al. (2021) who found a positive association between exposure to air pollutants and inflammatory mediators as a pathway, through which airborne particulate matter may lead to short-term increases in cardiac risk.

Furthermore, estimation of hematological parameters usually reflects the general health of the experimental animals. The present study showed insignificant changes in RBC count, Hb content, and Hct% in both the exposed young and adult groups compared to their respective control groups, confirming recent study of Anyabolu et al. (2021) who reported that inhalation of mosquito coil smoke was not anemic. Moreover, obtained data also showed significant increase in serum EPO hormone in both exposed groups. Idowu et al. (2013) suggested that the increase in EPO hormone is responsible for stimulating bone marrow to produce RBCs to overcome hypoxia. In turn, the maintenance of normal levels of hematological parameters in the present study can be attributed to the increase of EPO hormone in the exposed groups.

The present study revealed that young rats were more vulnerable to the harmful effects of mosquito coil smoke that appeared in most biochemical and histopathological investigations compared to adult rats. Buka et al. (2006) attributed this to the rapid development of the cardiopulmonary system in young age that may make them more susceptible to injury and inflammation caused by pollutants. Other studies explained that young age has more immature immune system resulting in rapid inflammatory responses and autoimmune injuries (Burroughs Peña and Rollins 2017; Wang et al. 2019). In addition, Ntarladima et al. (2019) explained the sensitivity of young age to air pollutants as they breathe in more air per unit of body weight and consequently more air pollutants. This could be also supported by the pronounced decrease in CYP450 expression in the control young group compared to control adult group, referring to low detoxifying ability in young age, making them more vulnerable to injury. This agrees with Xu et al. (2019) who observed the same impact on the liver of rats. Subsequently, the heart of young exposed rats was more susceptible to hazards than the adult.

## Conclusion

Various pollutants, including particulate matter, volatile organic compounds, and formaldehyde, were present in the generated smoke of mosquito coils that contained 0.05% meperfluthrin which achieved extremely contaminated standards within the first hour of exposure. Severe cardiac consequences include significant changes in biochemical parameters, inflammation, and apoptosis. The young rats were more vulnerable to negative consequences than adults. This study recommended a controllable usage of synthetic pyrethroid insecticides as their indoor use will result in unbearable side effects on the heart as shown in the present study. Moreover, additional research is advised and warranted for a future effective therapy to further elaborate on the reduction of harmful effects of mosquito coil smoke.

## Data Availability

All data generated and analyzed are available upon request.
